# Reduced Margin Stereotactic Body Radiation Therapy for Early Stage Non-Small Cell Lung Cancers

**DOI:** 10.7759/cureus.8618

**Published:** 2020-06-14

**Authors:** Mayank Patel, Tyler Colvin, Robert Spencer Kirkland, Samuel Marcrom, Michael Dobelbower, Sharon A Spencer, Drexell H Boggs, Richard Popple, Sui Shen, Benjamin Wei, Andrew McDonald

**Affiliations:** 1 Radiation Oncology, University of Alabama at Birmingham School of Medicine, Birmingham, USA; 2 Internal Medicine, University of Alabama at Birmingham School of Medicine, Birmingham, USA; 3 Cardiothoracic Surgery, University of Alabama at Birmingham School of Medicine, Birmingham, USA

**Keywords:** sbrt, stereotactic body radiation therapy, local control, reduced margin, lung cancer, fiducial marker, histology, 5-mm isotropic margin

## Abstract

Purpose

Our study reports the clinical outcomes of patients treated with 5-mm isotropic margin, fiducial-guided stereotactic body radiation therapy (SBRT) for early stage non-small cell lung cancer (NSCLC). We also sought to assess the effect of histological subtype on local control.

Methods

We retrospectively reviewed the charts of all patients treated with SBRT for NSCLC between 2007 and 2017 at our institution. All patients who had implanted fiducial markers, planning target volume (PTV) margins of 5 mm or less, early stage disease (T1-T2, N0), and at least one follow-up CT were included in this analysis. Estimates of local control were generated using the Kaplan-Meier method, and differences between survival curves were assessed using the log-rank test.

Results

A total of 152 patients met the inclusion criteria for this analysis, with a median follow-up of 27.9 months. Patients received 54 Gy in three fractions for peripheral tumors and 48-52.5 Gy in four to five fractions for central tumors. NSCLC histology was adenocarcinoma in 69 (45.4%) cases, squamous cell carcinoma in 65 (42.8%) cases, and other or non-subtyped in 18 (11.8%) cases. Across the entire cohort, the two-year estimate of local control was 95.1%. When histology was considered, the two-year estimate of local control among patients with adenocarcinoma was 95.6% as compared with 85.0% for patients with other subtypes (p=0.044).

Conclusions

Fiducial-guided, isotropic 5-mm PTV margin for thoracic SBRT did not compromise local control compared with historical standards. In this series, patients with adenocarcinoma experienced improved local control compared with squamous cell carcinoma.

## Introduction

Stereotactic body radiation therapy (SBRT) is a rapidly evolving radiation therapy technique that is showing promising results for the treatment of early stage non-small cell lung cancer (NSCLC). SBRT is typically used for the treatment of patients who refuse surgical resection or have comorbidities rendering them poor surgical candidates [1]. Despite being used primarily in a patient population whose demographics are skewed toward greater comorbidity and decreased life expectancy, SBRT has shown impressive rates of local control (LC), which, in turn, has reduced lung cancer specific mortality [2-4].

SBRT delivers focal treatment, but radiation can still harm the surrounding healthy tissue. Early lung SBRT techniques used large internal target volume (ITV) margins with treatment delivered over the entire respiratory cycle [5]. Planning tumor margins were also asymmetric to account for increased tumor motion in the superior-inferior direction. Larger target volume expansions result in increased radiation exposure to normal lung parenchyma. At our institution, we used a combination of respiratory-gated treatment delivery and fiducial guidance in order to reduce both ITV and planning target volume (PTV) margins and consequently normal lung dose exposure. The ability to gate treatment delivery to a window around end-expiration allows for a significant reduction in ITV size [6]. Implantation of fiducial markers offers improved accuracy in tumor dose delivery with real-time target monitoring, which makes it possible for reduced PTV margins [6]. The purpose of this report is to describe the LC rates for early stage NSCLC treated with the reduced margin SBRT technique used at our institution. We hypothesized that LC would be similar to previously published reports of lung SBRT.

## Materials and methods

Inclusion criteria

The records of all patients who received SBRT for NSCLC at our institution from 2007 to 2017 were reviewed. All patients who had fiducial markers placed, underwent SBRT with planning target volume (PTV) margins no larger than 5 mm, had early stage disease (T1-T2, N0), and had at least one follow-up CT were included in this study. Gating techniques were available at the study institution in 2007 and were used unless contraindicated. This study was reviewed and approved by the Institutional Review Board at our institution.

Treatment

Following a histological diagnosis of NSCLC, patients were reviewed by a multidisciplinary team. Once the decision was made to proceed with lung SBRT, patients underwent placement of fiducial markers by either navigational bronchoscopy or CT-guided transthoracic needle placement. Either two or three fiducial markers were placed within or adjacent to the tumor. A four-dimensional (4D) CT simulation was performed for all patients using the Varian Real-Time Position Management system (Varian Medical Systems, Palo Alto, CA, USA) on a Philips Brilliance Big Bore CT scanner (Koninklijke Philips N.V., Amsterdam, the Netherlands). Target motion magnitude in principal axis was assessed using 10-phase 4DCT. Phases with the residual target motion within 5 mm around the end of expiration were selected and used to create average and maximum intensity projection (MIP) data sets. The gross tumor volume (GTV) was delineated using the MIP and verified with individual phase images selected. The GTV volumes were saved to planning CT data set using average data sets. The decision to use respiratory gating was made if tumor motion was more than 5 mm during respiration. In patients with irregular breathing, all phases of breathing were included to delineate a GTV. Organs-at-risk (OARs) were delineated using the average data set. Since the GTV was delineated using the MIP based on selected phase images of 4DCT scan, no additional ITV margin was added. The PTV was generated as asymmetric 5-mm geometric expansion around the GTV.

PTVs that were not within 2 cm of the proximal bronchial tree and not in direct contact with the chest wall were generally prescribed a dose of 54 Gy in three fractions, and doses of 48-52.5 Gy in four to five fractions were prescribed in the remaining cases. Inverse planning with heterogeneity correction was used in all cases, and the most common beam arrangement was volumetric modulated arc therapy (VMAT) with two axial arcs. OAR constraints were in agreement with those published in the National Comprehensive Cancer Network (NCCN) guidelines; for tumors in close proximity to an OAR, the OAR dosimetry goals were prioritized above the gradient index.

Treatments were delivered on non-consecutive days by either a Varian Clinax iX, Varian TrueBeam STX, or Varian Edge linear accelerator using 6- to 15-MV photons. A combination of gated orthogonal radiographs and cone beam CT (CBCT) were used for pretreatment alignment, with CBCT designated as the primary alignment technique. CBCT was used for alignment to fiducial markers along with alignment assistance from the surrounding soft tissues, paying particular attention to central OAR when in relevant proximity. The Real-Time Positioning Management system was used to gate the treatment delivery during only the planned portion of end-expiration. The positions of the implanted fiducial markers were monitored throughout treatment delivery using triggered kV orthogonal radiographs to ensure that fiducials were within their 5-mm contours. The decision to put beam “on hold” was made by the treatment team when the fiducials were outside 5-mm contours and until they returned to their original position.

Toxicity evaluation and follow-up

Our routine post-treatment assessment consists of acute toxicity evaluation on the final day of treatment and at one- and three-month clinic visits. A baseline chest CT with IV contrast was generally performed four to six weeks after treatment. Following these two appointments, patients returned every 3 to 6 months for three years and then every 6 to 12 months thereafter. Each follow-up visit included a history and physical examination as well as a chest CT, with the use of IV contrast at the discretion of the treating physician. Additional imaging such as positron emission tomography (PET) was performed if CT findings were ambiguous.

Endpoint definitions and statistical methods

Local failure was defined as definite tumor recurrence or enlargement within 1 cm of the previously treated PTV on surveillance imaging, and pathological confirmation of viable tumor was obtained when feasible. This broad definition of local failure was consistent with prior Radiation Therapy Oncology Group (RTOG) protocols and would have included marginal failures [7]. LC was defined as the absence of local failure and was measured from the CBCT from the first fraction of SBRT. Patients without an evidence of local failure were censored from survival analyses at the time of most recent imaging. Overall survival was measured from the time of first SBRT fraction until the date of death. Surviving patients were censored at the time of most recent clinical encounter. Estimates of LC and overall survival were generated using the Kaplan-Meier method and differences in survival curves were assessed using the log-rank test. Univariate Cox proportional hazards models were used to assess the effect of potential variables on LC and to generate hazard ratios (HRs). Multivariable analysis was not performed given the small number of local failures. All statistical analysis was performed using SPSS Version 24.1 (IBM Corp., Armonk, NY, USA).

## Results

Patient characteristics and overall survival

**Figure 1:**
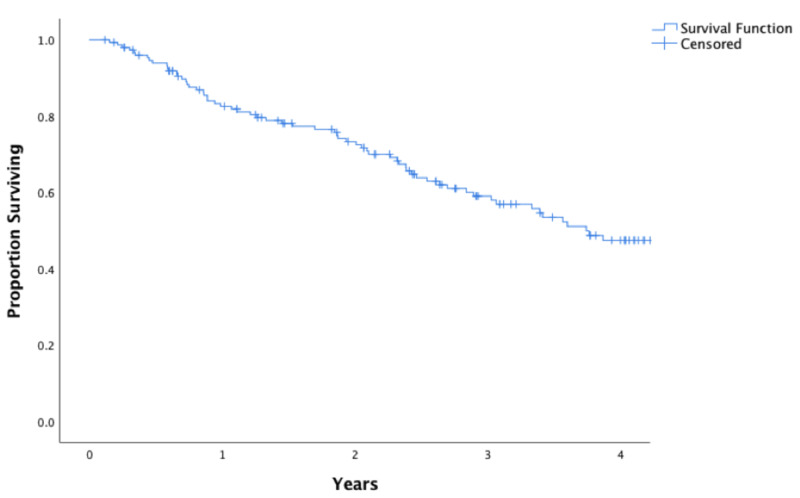
Kaplan-Meier estimates of overall survival The Kaplan-Meier estimate of overall survival across the entire cohort is 78.1% at two years.

**Figure 2:**
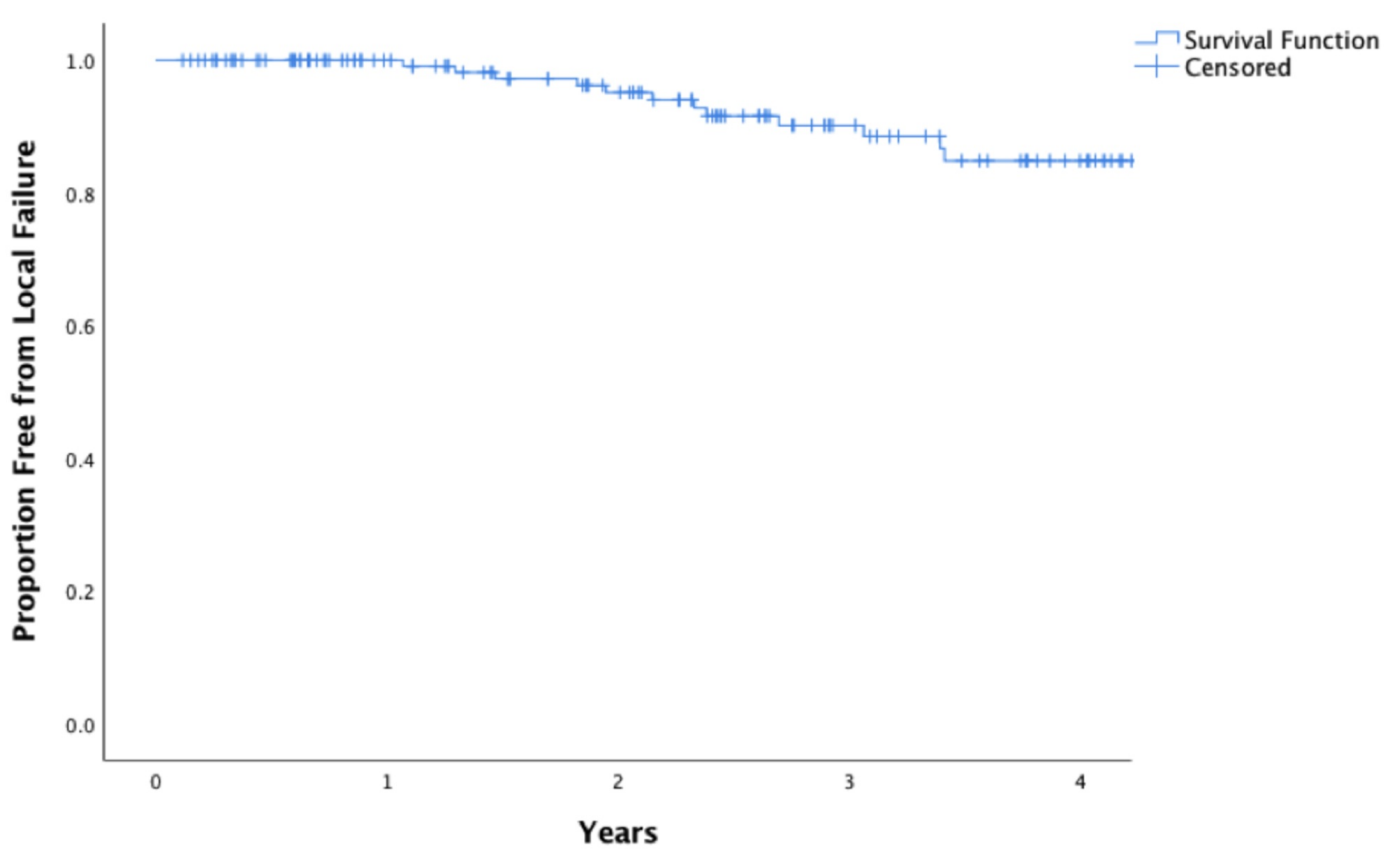
Kaplan-Meier estimates of local control The Kaplan-Meier estimate of local control across the entire cohort is 95.1% at two years.

A total of 361 patients were treated with SBRT between 2007 and 2017, of whom 152 were included in this study. The most common reasons for excluding patients were fiducial markers not placed (125 patients), PTV margins > 5 mm (20 patients), lack of follow-up imaging (31 patients), or advanced tumor staging (25 patients). A description of the baseline demographic, disease, and treatment characteristics is given in Table 1. The median radiographic follow-up was 1.85 years (interquartile range: 0.11-8.36 years). The Kaplan-Meier estimate of overall survival at two years was 78.1% and the median survival was 3.7 years (Figure 1). Across all patients, the Kaplan-Meier estimate of LC at two years was 95.1% (Figure 2).

**Table 1:** Patient and Tumor Characteristics RUL, right upper lobe; RML, right middle lobe; RLL, right lower lobe; LUL, left upper lobe; LLL, left lower lobe; NSCLC, non-small cell lung cancer

Characteristic	Number of Patients	Percent of Patients
Gender		
Male	83	54.6%
Female	69	45.4%
Ethnicity		
White	131	86.2%
Black	19	12.5%
Other	2	1.3%
Age		
≥65 years	121	79.6%
<65 years	31	20.4%
Location of tumor		
Central (near the proximal bronchial tree)	46	30.2%
Peripheral	106	69.7%
Tumor size		
≥2.5 cm	85	55.9%
<2.5 cm	67	44.1%
Smoking history		
Positive history	145	95.4%
Never smoker	7	4.6%
≥20 pack-years	131	90.3%
<20 pack-years or unknown length	14	9.7%
Lobe		
RUL	45	29.6%
RML	10	6.6%
RLL	24	15.8%
LUL	55	36.2%
LLL	18	11.8%
Histology		
Adenocarcinoma	69	45.4%
Squamous cell carcinoma	65	42.8%
Non-differentiated NSCLC	18	11.8%
Local recurrence		
Yes	12	7.9%
No	140	92.1%

**Figure 3:**
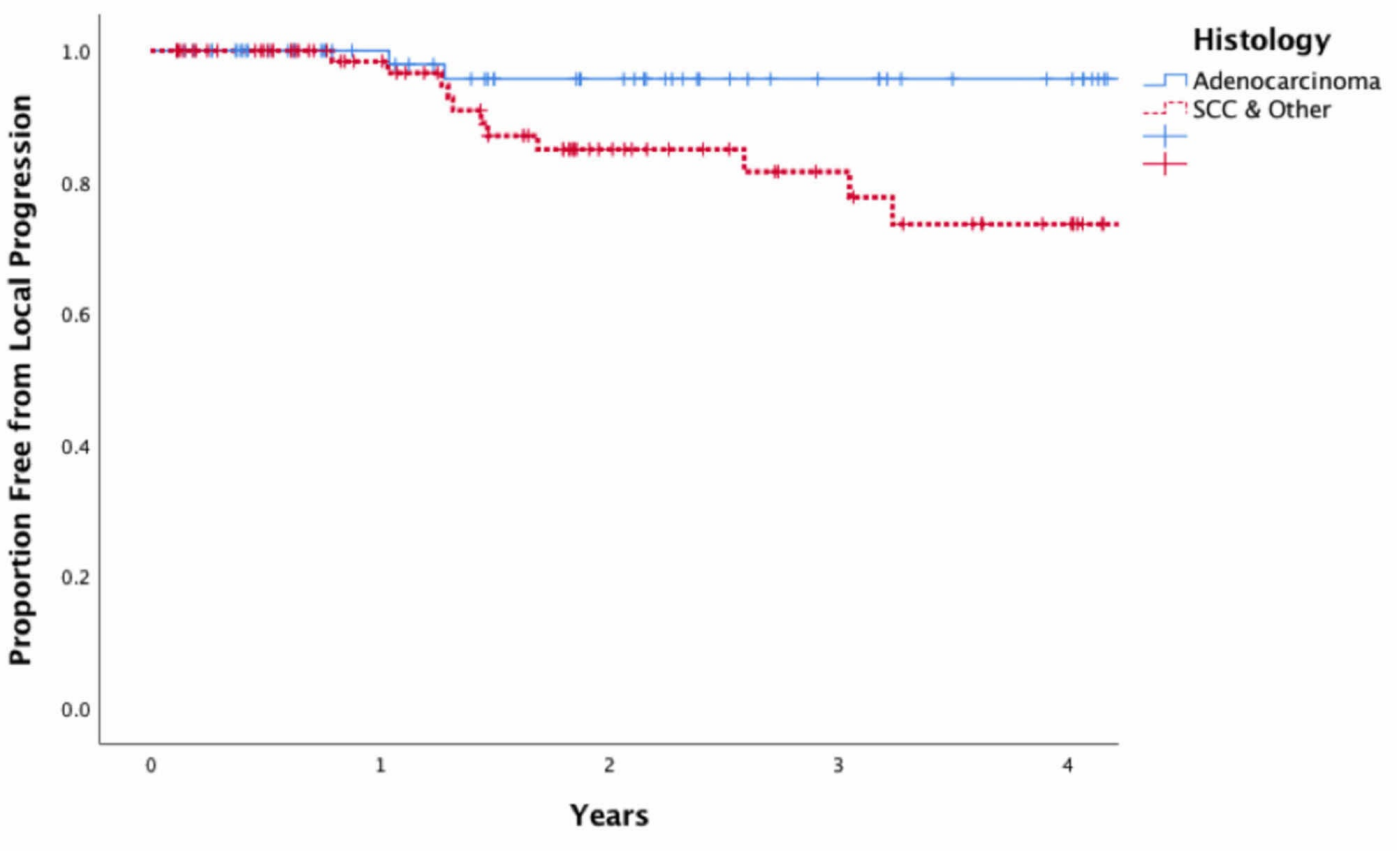
Kaplan-Meier estimates of local control by histology The Kaplan-Meier estimates of local control were stratified by histology. At two years, the estimate of local control was 95.6% among patients with adenocarcinoma as compared with 85.0% among patients with other subtypes (p=0.044). SCC, squamous cell carcinoma

The results of the univariate Cox proportional hazards models assessing the effects of patient, tumor, and treatment variables on LC are presented in Table 2. In particular, increased pack-years of smoking was associated with an increased hazard of local recurrence when analyzed as a continuous variable (HR=1.011; 95% CI: 0.996-1.027) as was non-adenocarcinoma histology (HR=3.683; 95% CI: 0.792-17.130). Figure 3 presents the Kaplan-Meier estimates of LC stratified by histology. At two years, the estimate of LC was 95.6% among patients with adenocarcinoma as compared with 85.0% among patients with other subtypes (p=0.044).

**Table 2:** Cox Hazard Ratio for Local Recurrence

Variable	Hazard Ratio	Lower 95% CI	Upper 95% CI	p-Value	Significance
Central location	1.302	0.352	4.820	0.692	No
Adenocarcinoma	3.683	0.792	17.130	0.076	No
Pack-years	1.011	0.996	1.027	0.156	No

## Discussion

Lung cancer radiotherapy often uses large ITV and PTV expansions in order to account for motion during respiration. Additional techniques can ensure accurate treatment delivery and eliminate the need for these expansions. Our retrospective study reports LC rates in patients treated with the reduced margin SBRT technique used at our institution. Our institutional technique involves respiratory-gated planning and treatment delivery with fiducial guidance. Respiratory gating reduces radiation exposure to healthy tissue by applying irradiation only during the end-expiratory phases. Recent studies have shown that respiratory gating often reduces PTVs by more than 30%, and some studies have shown greater than 50% reduction [8]. Minimizing the treated volume reduces the amount of radiation to surrounding healthy tissues and theoretically limits toxicities. Fiducial marker the placement by either navigational bronchoscopy or CT-guided transthoracic needle placement allows for improved tumor localization. The fiducial acts as a reference marker, which can be viewed prior to and during treatment. This increases confidence in precision of treatment delivery when used in combination with respiratory gating, as the fiducials are used to confirm target position for every respiratory-gated treatment window. Fiducial placement is seen as a relatively safe procedure, though pneumothorax and pulmonary hemorrhage are known complications [9-10]. The 5-mm isotropic margins seen in our study differ from standard margins seen in larger trials. For example, RTOG 0236 treated patients with 10 mm in the craniocaudal dimension and 5 mm in the axial dimension. Tumor position during respiration was confirmed with 4DCT imaging in their trial, and patients with tumor movement outside of the PTV expansions were treated with abdominal compression, respiratory gating, or breath-holding [5]. Fiducial placement was not incorporated in the treatment of these patients [5]. As stated above, our study is able to treat with 5-mm isotropic margins and reduce PTV expansions in the craniocaudal dimension due to our institutional technique of respiratory gating and fiducial placement.

Our retrospective study included 152 patients who met the inclusion criteria of fiducial placement, 5-mm symmetrical margins, and at least one follow-up CT post-treatment. We observed a 95.1% estimate of LC rate at two years post-SBRT, with a median follow-up time of 27.9 months. In a recent meta-analysis of more than 7,000 patients who underwent SBRT for early-stage NSCLC, the one- and three-year estimates of LC were 96.3% and 87.8%, respectively. Mean follow-up among these patients was 27.3 months [11]. The difference in LC rates between our study and the meta-analysis was minimal. While our study treated patients with both central and peripheral tumors, several studies in the meta-analysis (RTOG 0236 and 0915) solely treated patients with peripheral tumors [5,12]. Higher radiation doses can be used for peripheral tumors and could have potentially led to higher rates of LC. Histologically, our study comprised 42.8% squamous cell carcinoma and 45.4% adenocarcinoma, which is a higher percentage of squamous cell carcinoma than the national average of 30% [13,14]. Squamous cell carcinoma is traditionally less responsive than adenocarcinoma to radiation therapy and is more prone to local recurrence [14,15]. Histology was not recorded in the meta-analysis.

Series using PTV of 5 mm or less are scarce as most studies use larger margins in the craniocaudal dimension. One relevant study, Lischalk et al., evaluated the long-term outcomes of reduced margin SBRT with fiducial tracking for inoperable stage I NSCLC. In that study, there were 61 patients treated with 5-mm symmetric margins using fiducial placement. The Kaplan-Meier method estimated LC rates to be 91.1% and 87.6% at three and five years, respectively. There were four instances of local failure that occurred at an average of 2.6 years [16]. The high rates of LC could partially be attributed to the low proportion of squamous cell carcinoma patients in that study (26%). In that study, 49% of the patients were of the adenocarcinoma cell type. In our study, there was a near significant (p=.076) difference in rates of LC among patients with non-adenocarcinoma and adenocarcinoma cell type. The rates of LC seen in Lischalk et al.’s study are comparable to our study, which observed 89.8% at two years. The findings of our study further suggest that small margin SBRT does not compromise tumor control but is shown in a larger cohort size than previous studies.

The most common histologies of NSCLCs are adenocarcinomas and squamous cell carcinomas. Adenocarcinoma and squamous cell carcinomas account for the majority of NSCLCs (around 50% and 30%, respectively), with the remaining 20% recognized as undifferentiated or other less common histologies [13-14]. Our study included 69 patients with adenocarcinoma, 65 patients with squamous cell carcinoma, and 18 with undifferentiated histology. Squamous cell carcinoma or undifferentiated histology was found to be a predictor for failure of LC. Kaplan-Meier estimates showed an LC rate of 85.0% at two years for non-adenocarcinoma compared with a 95.6% rate for adenocarcinoma. Our study had a higher proportion of squamous cell carcinoma compared with the national average [13]. Thus, the LC rates may be affected by the relatively higher proportion of squamous cell carcinoma. We then looked at additional SBRT studies addressing the effect of histology on LC. Woody et al. (n=740) showed that squamous cell carcinomas were two times more likely to fail locally as well as being more resistant to radiation than other non-small cell histologies [13]. The overall LC rates in their study were 88.2% at three years, with 81% for squamous cell carcinoma. The median follow-up time was 15.5 months [13]. These numbers are consistent with the LC rates and follow-up reported in our study.

An important future area of interest with reduced margin SBRT will be the effect on toxicities. While acute and chronic toxicities are not commonly seen in SBRT (<10%) [17], complications include esophagitis, atelectasis, vascular injuries, and radiation pneumonitis acutely [18]. Further data collection will be performed for patients in our study to determine the rates of acute and chronic toxicities. Another area of focus will be the correlation of tumor position with high-grade toxicities. Patients with centrally located tumors are at an increased risk of high-grade toxicities [19], which can likely be attributed to the proximity to vital organs. We would expect to see higher rates of grade III+ toxicities in the patients with centrally located tumors in our cohort. In summary, while relatively uncommon, acute and chronic toxicities can occur in patients receiving radiation therapy. Reduced margin SBRT may potentially lead to a decrease in these toxicities due to the smaller amount of normal tissue affected [18].

The primary limitations of this study are the retrospective design, limited follow-up, and heterogenous patient population. Because this was a retrospective study, many patients were lost to clinical and radiographic follow-up. We attempted to minimize this effect by requiring at least one follow-up CT imaging as inclusion criteria. Although the inclusion criteria of this study were broad, the exclusion of patients who did not have fiducial markers placed may be a source of bias.

The major concern for reducing margin size in SBRT for NSCLC is the potential for leaving tumor cells untreated. The incorporation of respiratory gating and fiducial placement allows for greater treatment precision. We did not observe that reducing the margins had an effect on LC. An important future direction is continued follow-up of this cohort to assess radiation toxicity following 5-mm isotropic margin treatment. Limiting these toxicities will likely be beneficial to minimize the burden placed on the patient. While this remains a preliminary study, the results were encouraging for LC of early stage NSCLC treated with 5-mm isotropic SBRT.

## Conclusions

SBRT offers and continues, as in this study, to demonstrate high LC rates for patients with early-stage NSCLC. With fiducial guidance and reduced treatment margins, this study reports high LC rates that are comparable with previously described series with larger treatment margins. With reduced treatment margins, more patients with early stage NSCLC should be considered candidates for this highly effective treatment modality, particularly for more centrally located tumors with nearby healthy organs at risk of toxicity.
